# Serum proteomics and machine learning identify PSMD11 as a prognostic biomarker in severe fever with thrombocytopenia syndrome

**DOI:** 10.3389/fimmu.2025.1693946

**Published:** 2025-11-05

**Authors:** Chenxi Zhao, Ziruo Ge, Ranran Wang, Yanli Xu, Tingyu Zhang, Zhouling Jiang, Lu Liu, Ling Lin, Zhihai Chen

**Affiliations:** ^1^ National Key Laboratory of Intelligent Tracking and Forecasting for Infectious Diseases, Beijing Ditan Hospital, Capital Medical University, Beijing, China; ^2^ Department of Infectious Diseases, Yantai Qishan Hospital, Yantai, Shandong, China

**Keywords:** severe fever with thrombocytopenia syndrome, proteomics, machine learning, PSMD11, SHAP

## Abstract

**Background:**

Severe fever with thrombocytopenia syndrome (SFTS) is an emerging tick-borne viral disease associated with high mortality. This study aimed to characterize serum proteomic signatures linked to adverse outcomes and to identify prognostic biomarkers with potential translational value for patient management.

**Methods:**

Serum samples from 55 survivors, 32 non-survivors, and 10 healthy controls were analyzed by data-independent acquisition–based proteomics. Differential abundance analysis, Gene Ontology (GO) enrichment, Kyoto Encyclopedia of Genes and Genomes (KEGG) pathway analysis, and protein–protein interaction (PPI) network analyses with Markov clustering were conducted to characterize disease-associated proteins. XGBoost and Random Forest machine learning models were applied to prioritize candidate biomarkers, and discriminative performance was evaluated by the receiver operating characteristic (ROC) curve. Spearman correlation analyses were further used to examine associations between candidate proteins, clinical laboratory indicators, and viral load.

**Results:**

Non-survivors exhibited 642 differentially abundant proteins (DAPs) compared with survivors. Functional enrichment and PPI network analyses revealed a proteasome-centered module overrepresented in non-survivors. XGBoost and Random Forest consistently prioritized four candidate biomarkers (PSMD11, IL1RL1, PSMC4, and IFIH1) with areas under the ROC curve of 0.847, 0.847, 0.843, and 0.791, respectively. PSMD11 emerged as the strongest predictor of adverse outcome and showed strong correlations with markers of organ injury and dysfunction such as lactate dehydrogenase (*r* = 0.77), thrombin time (*r* = 0.76), aspartate aminotransferase (*r* = 0.75), hydroxybutyrate dehydrogenase (*r* = 0.74), viral load (*r* = 0.63), and platelet count (*r* = −0.57) (all *p* < 0.001).

**Conclusions:**

This study identified a proteasome-centered signature associated with adverse outcomes in SFTS, with PSMD11 emerging as a key prognostic biomarker. Its strong correlations with viral load and multi-organ injury support potential utility for early risk stratification and prognostic prediction, while also providing mechanistic insights into disease progression and a foundation for future translational research and therapeutic development.

## Introduction

Severe Fever with Thrombocytopenia Syndrome (SFTS) is an emerging tick-borne infectious disease caused by *Bandavirus dabieense* (family *Phenuiviridae*) (1), commonly known as SFTS virus (SFTSV), which was first identified in China in 2009 (2). Since then, SFTS has been increasingly reported across East and Southeast Asia, including South Korea, Japan, Vietnam, Thailand, and Pakistan, with both annual cases and geographic range continuing expand (3–7). SFTS is characterized by acute high fever, thrombocytopenia, leukopenia, hemorrhagic manifestations, and gastrointestinal symptoms, and can progress to multiorgan dysfunction and death in severe cases (8). According to a recent meta-analysis, the overall pooled case-fatality rate was 7.80% (95% CI, 7.01–8.69%) (9), emphasizing the considerable mortality burden that persists despite improvements in supportive care. Favipiravir was approved for the treatment of SFTS for the first time in Japan in June 2024 (10). However, randomized evidence remains limited and there are still no globally licensed vaccines or widely accessible targeted antivirals for SFTS (11). The WHO’s 2024 prioritization classified *Phenuiviridae* as high risk for Public Health Emergency of International Concern (PHEIC) and identified *Bandavirus dabieense* as a priority pathogen for research and development (12). These underscore the urgency of elucidating SFTS pathogenesis and accelerating biomarker-informed strategies for prognosis and therapeutic development.

Accumulating evidence indicates that dynamic changes in circulating biomarkers—including interleukin-6 (IL-6), IL-10, interferon-α (IFN-α), lactate dehydrogenase (LDH), ferritin, and C-C motif chemokine 20 (CCL20)—track with disease severity and mortality risk, reflecting the importance of host–pathogen interactions (13–15). High-throughput proteomics has accelerated both fundamental and applied research on emerging and re-emerging viral pathogens (16). By enabling quantitative assessment of protein abundance, proteomics provides direct insights into disease progression (17). Serum and plasma proteomic profiling has been widely applied to SARS-CoV-2, revealing host–pathogen interactions, elucidating the molecular mechanisms underlying COVID-19 pathology, and identifying candidate biomarkers for risk stratification and therapeutic development (18–20). Machine learning (ML) has become an essential tool in clinical and translational research, facilitating the identification of diagnostic, prognostic, and predictive biomarkers (21). A recent study developed a machine learning model based on circulating protein signatures that accurately predicted COVID-19 severity, highlighting the value of proteomics-driven ML strategies for clinical risk assessment (22). Integrating proteomics with ML approaches offers a promising strategy to generate clinically relevant insights into emerging infectious diseases (23). Nevertheless, applications of such integrative analyses to SFTS remain scarce.

In this study, our objective was to integrate serum proteomics with interpretable ML to delineate molecular signatures of SFTS, identify candidate biomarkers with translational potential, and illuminate disease-relevant pathways. By providing a systems-level view of host–pathogen interactions, our work aims to improve risk stratification and inform the development of targeted interventions, thereby contributing to better clinical management of SFTS.

## Methods

### Study design and patients

This prospective cohort study was conducted at Yantai Qishan Hospital between May and September 2024. A total of 87 patients with laboratory-confirmed SFTS were enrolled at admission, comprising 55 survivors and 32 non-survivors. SFTS diagnosis was confirmed by real-time quantitative reverse transcription polymerase chain reaction (RT-qPCR) detection of SFTSV RNA. Inclusion criteria were age ≥ 18 years and admission within 7 days of symptom onset. Exclusion criteria were: co-infection with other acute or chronic infections; receipt of antitumor therapy; inability to obtain required samples; or incomplete essential clinical data. Ten age- and sex- matched healthy controls (HC) with negative SFTSV tests were included. The primary endpoint was defined as either recovery with discharge or in-hospital death.

The study was conducted in accordance with the principles of the Declaration of Helsinki and was approved by the Ethics Committee of Beijing Ditan Hospital, Capital Medical University (No. DTEC-KY2022-022). Written informed consent was obtained from all participants or their relatives.

### Clinical sample collection and definitions

For patients with SFTS, peripheral venous blood was collected within 24 hours of hospital admission using silica-coated clot activator tubes without gel separators. A total of 97 serum samples were obtained from 87 patients with SFTS and 10 healthy donors. Samples were centrifuged at 2,000 × g for 10 minutes within 2 hours of collection to ensure complete serum separation. The resulting serum was aliquoted and stored at −80°C until analysis. Serum viral load was measured using a commercial RT-qPCR kit (Daan, Guangzhou, China) according to the manufacturer’s instructions.

The acute phase, characterized by high fever and systemic symptoms, was defined as days 1–7 from symptom onset (24).

### Data-independent acquisition proteomics

Serum samples were processed using a standardized DIA proteomics workflow. Briefly, 15 μL of serum was used as the starting volume and combined with 75 μL of Buffer 2 from the OmniProt Kit (OmniProt, China), followed by centrifugation at 4,000 rpm for 2 minutes at room temperature. The supernatant was incubated with 90 μL of depletion reagent at 32°C for 1 hour with gentle agitation (220 rpm) to remove high-abundance proteins, followed by centrifugation at 17,000 × g for 10 minutes. The pellet was washed twice with Buffer 3 and re-centrifuged under the same conditions. The resulting proteins were solubilized in lysis buffer (6 M urea, 2 M thiourea), reduced with tris (2-carboxyethyl) phosphine (0.2 M, 4 μL, 32°C, 30 minutes), and alkylated with iodoacetamide (0.8 M, 4 μL, 32°C, 30 minutes). Proteins were digested overnight with sequencing-grade trypsin at 32°C with gentle shaking (220 rpm). The reaction was quenched with 30 μL of 10% trifluoroacetic acid, and peptides were centrifuged at 17,000 × g for 10 minutes. The supernatant was transferred to a new tube, and the pH was adjusted to 2. Peptides were desalted using SOLAμ solid-phase extraction plates (Thermo Fisher Scientific, San Jose, USA) according to the manufacturer’s instructions. The eluates were vacuum dried at 40°C, reconstituted in 0.1% formic acid, and quantified at 280 nm using a NanoDrop spectrophotometer. Peptide concentrations were adjusted to 0.2 μg/μL to ensure equal loading across all samples. For DIA analysis, 1 μL of each digest (equivalent to 200 ng of peptides) was injected into a Vanquish Neo UHPLC system coupled to an Orbitrap Astral mass spectrometer (Thermo Fisher Scientific, San Jose, USA). Peptides were separated using a 24-minute liquid chromatography gradient. Raw DIA data were processed with DIA-NN (version 1.8.1) in single-pass (library-free) mode for protein identification and quantification.

Samples were analyzed in 11 experimental batches under strictly standardized procedures, including identical reagent lots, uniform sample preparation, and consistent instrument calibration. Each batch contained one pooled quality control (QC) sample and one biological replicate sample to monitor intra- and inter-batch consistency. The pooled QC sample was prepared by mixing equal aliquots from all experimental samples.

### Proteomic data analysis

Proteins were identified and quantified using library-free DIA-NN searches against a human reference database. Proteins with more than 70% missing values across all samples were excluded prior to downstream analysis, and the remaining missing values were imputed using the k-nearest neighbors (KNN) algorithm after quality control. Outliers were defined as values exceeding three times the interquartile range (IQR) above the upper quartile or below the lower quartile. Protein intensities were log_2_-transformed and Z-score normalized. Differential protein abundance was analyzed using the limma package in R, applying moderated t-statistics with empirical Bayes shrinkage. *p*-values were adjusted using the Benjamini-Hochberg method to control the false discovery rate (FDR). Differentially abundant proteins (DAPs) were defined as FDR < 0.05 and a fold change (FC) > 2 or < 0.5 (i.e., absolute log_2_FC > 1). Gene Ontology (GO) annotation categorized DAPs into biological process (BP), cellular component (CC), and molecular function (MF). Kyoto Encyclopedia of Genes and Genomes (KEGG) pathway enrichment was performed to identify significantly enriched pathways among DAPs. Protein–protein interaction (PPI) networks via the search tool for retrieval of interacting genes/proteins (STRING) database (version 12.0) to explore interactions among proteins, a combined score > 0.7 was applied to ensure a high-confidence interaction threshold in the identified interactions and visualized in Cytoscape (version 3.10.3).

### Machine learning algorithms

Discriminative proteins among the DAPs were identified using two supervised machine learning algorithms, eXtreme Gradient Boosting (XGBoost) and Random Forest. The XGBoost model was trained using a 10-fold cross-validation procedure with a fixed random seed to ensure reproducibility. The model was optimized for binary classification based on the log-loss objective function, and hyperparameters were tuned to balance model complexity and mitigate overfitting. Feature importance was evaluated using SHapley Additive exPlanations (SHAP), with the mean absolute SHAP value was used to rank the relative importance of DAPs. The Random Forest model was trained using the caret framework with 10-fold cross-validation and a fixed random seed to ensure reproducibility. Variable importance for each protein was determined by the mean decrease in the Gini index, averaged over folds and normalized for comparison across features.

### Statistical analysis

Clinical continuous variables were summarized as mean ± standard deviation (SD) or median (IQR) and compared with Student’s t-test or Mann–Whitney U test. Categorical variables were presented as counts (percentages) and compared using the χ² test or Fisher’s exact test. Multivariate patterns in proteomic data were explored by principal component analysis (PCA). Predictive performance of selected proteins was evaluated using receiver operating characteristic (ROC) and precision-recall (PR) analyses, reporting the area under the ROC curve (AUC) and the area under the PR curve (AUPRC) with 95% confidence intervals (CI). Additional performance metrics, including sensitivity, specificity, precision, accuracy, and F1 score, were visualized using radar charts. Correlations between selected DAPs and clinical laboratory parameters were analyzed using Spearman’s rank correlation and partial Spearman correlation controlling for age. All statistical analyses were performed in R software (version 4.4.2) using the following packages: limma, xgboost, SHAPforxgboost, randomForest, caret, pROC, PRROC, clusterProfiler, and ggplot2. Statistical significance was defined as *p* value (or FDR) < 0.05.

## Results

### Clinical characteristics of participants

The study enrolled 87 patients with SFTS, comprising 55 survivors (SA) and 32 non-survivors (NS), along with 10 age- and sex-matched HC. The SA had an average age of 65.38 ± 9.55 years, while the NS had significantly higher age of 70.6 ± 10.3 years (*p* = 0.023). No significant differences in sex distribution were observed between SA and NS (*p* = 0.868), or between SFTS patients and HC (*p* = 0.690). Compared with HC, patients with SFTS had significantly lower white blood cell (WBC) counts (*p* < 0.001), platelet counts(*p* < 0.001), and albumin levels(*p* < 0.001), as well as higher alanine aminotransferase (ALT) (*P* < 0.001), aspartate aminotransferase (AST) (*p* < 0.001), and creatinine levels (*p* < 0.001). Within the SFTS cohort, NS exhibited significantly lower platelet counts (*p <* 0.001) and albumin levels (*p* < 0.001), and higher AST (*p* < 0.001) and creatinine (*p* < 0.001) compared with SA. No significant differences were found in WBC counts (*p* = 0.403), hemoglobin (*p* = 0.758), mean platelet volume (MPV) (*p* = 0.867), or total bilirubin (TBil) (*p* = 0.060) between the survivor and non-survivor groups (**Table 1**).

**Table 1:** The baseline clinical and laboratory characteristics of the participants.

	Health control (n=10)	SFTS patients				*p* ^a^ value
		Total (n=87)	Survivor group (n=55)	Non-survivor group (n=32)	*p* ^b^ value	
Age (years)	61.4 ± 8.6	67.3 ± 10.1	65.38 ± 9.55	70.6 ± 10.3	0.023	0.068
Male, n (%)	5 (50)	33 (37.9)	20 (36.4)	13 (40.6)	0.868	0.690
WBC (^10^9^/L)	5.8 (5.2, 6.3)	2.1 (1.6, 3.1)	2.0 (1.5, 2.8)	2.1 (1.7, 3.1)	0.403	<0.001
Hemoglobin (g/L)	135.5 (130.0, 137.0)	143.0 (133.0, 152.0)	141.0 (133.0, 152.0)	144.5 (133.0, 151.8)	0.758	0.034
Platelet (^10^9^/L)	271.5 (237.5, 290.0)	65.0 (51.0, 82.0)	74.0 (61.0, 88.0)	51.5 (43.8, 65.8)	<0.001	<0.001
MPV(fL)	9.83 ± 0.66	10.46 ± 1.05	10.47 ± 1.13	10.44 ± 0.90	0.867	0.018
ALT (U/L)	14.0 (11.2, 25.1)	52.2 (34.2, 98.1)	43.3 (33.2, 90.2)	66.9 (46.0, 137.5)	0.059	<0.001
AST (U/L)	17.9 (15.2, 24.0)	122.3 (55.5, 213.7)	71.0 (49.1, 141.5)	170.2 (114.9, 456.9)	<0.001	<0.001
Albumin (g/L)	46.6 (45.6, 48.1)	34.9 (30.8, 37.4)	35.8 ± 4.2	31.8 ± 4.4	<0.001	<0.001
TBil (umol/L)	12.3 (11.0, 15.2)	9.3 (8.2, 11.3)	9.0 (7.9, 10.9)	10.5 (8.5, 14.7)	0.060	0.040
Creatinine (umol/L)	50.4 (47.0, 53.0)	74.0 (60.5, 94.2)	65.1 (57.0, 78.7)	99.9 (75.8, 152.8)	<0.001	<0.001

*p* ^a^: Comparisons between healthy controls and patients with SFTS.

*p* ^b^: Comparisons between survivors and non-survivors among patients with SFTS.

WBC, white blood cell. ALT, alanine aminotransferase. AST, aspartate aminotransferase. TBil, total bilirubin. MPV, mean platelet volume.

### Identification of differentially abundant proteins

**Figure 1:**
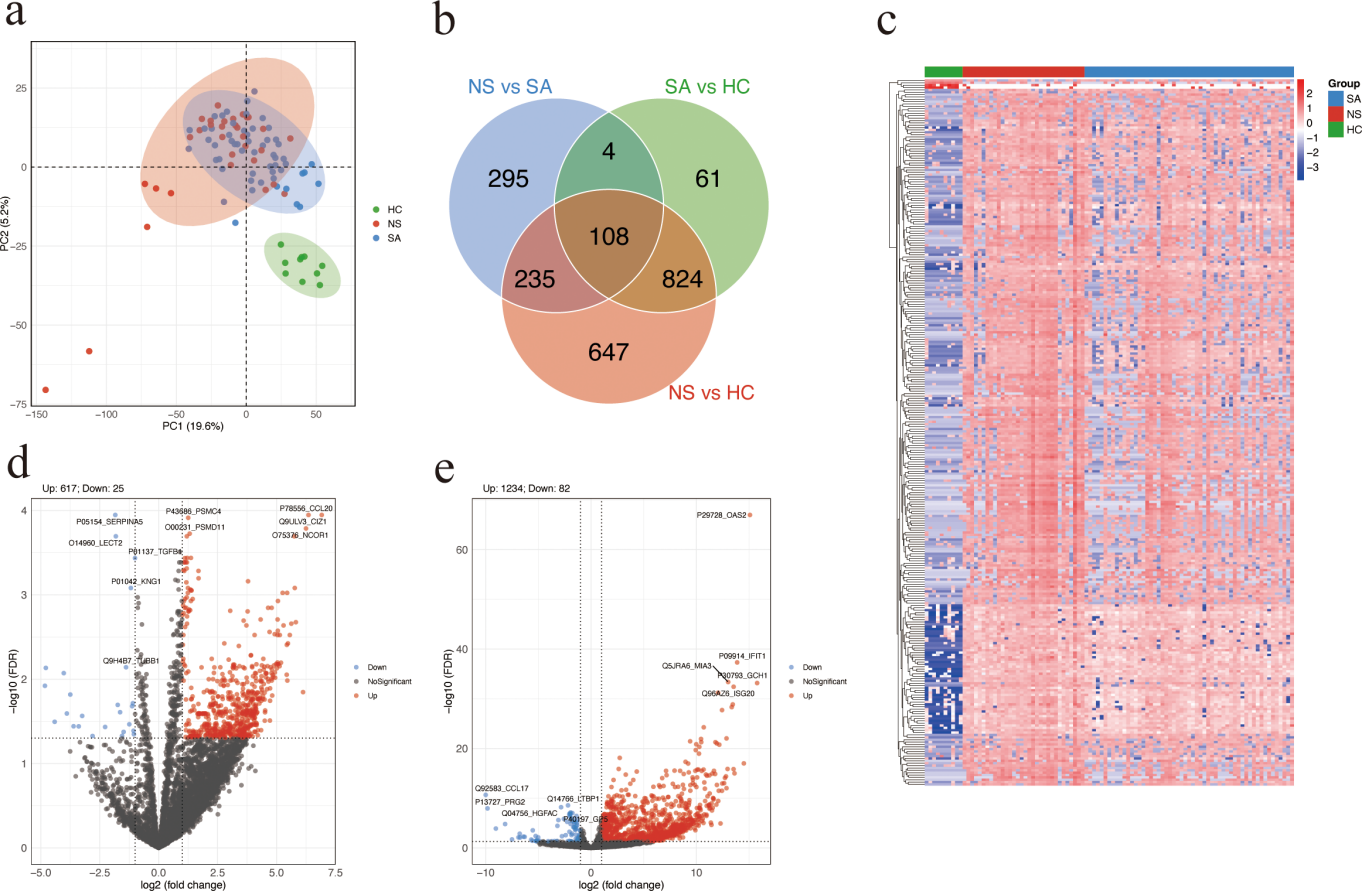
Differential protein abundance analysis. **(a)** Principal component analysis (PCA) plot of SA, NS, and HC based on proteomic profiles. Each dot represents a serum sample. **(b)** Venn diagram showing the overlap of DAPs among the three comparisons: NS *vs* SA, SA *vs* HC, and NS *vs* HC. **(c)** Heatmap showing the top 300 DAPs across the three groups. Higher and lower relative abundance levels are indicated in red and blue, respectively. **(d, e)** Volcano plot showing significant DAPs between NS and SA **(d)** or patients with SFTS and HC **(e)**. Red and blue dots represent significantly upregulated and downregulated proteins, respectively; the top five of each are labeled. Proteins with FDR (Benjamini-Hochberg adjusted) < 0.05 and absolute log_2_ (fold change) > 1 were considered significantly differentially. SA, survivors. NS, non-survivors. HC, healthy controls. DAPs, differentially abundant proteins.

A total of 97 serum samples were collected from 87 patients with SFTS and 10 HC. DIA proteomics quantified 5,541 proteins. PCA demonstrated distinct clustering among SA, NS, and HC (**Figure 1a**). Venn analysis identified 108 DAPs shared across NS *vs* SA, SA *vs* HC, and NS *vs* HC comparisons (**Figure 1b**). Hierarchical clustering of DAPs revealed a clear pattern, with upregulated proteins enriched in NS and downregulated proteins more prevalent in HC (**Figure 1c**). In total, 642 DAPs were identified between NS and SA, comprising 617 upregulated and 25 downregulated proteins. The top five upregulated DAPs in the volcano plot are CCL20, CDKN1A interacting zinc finger protein 1 (CIZ1), proteasome 26S subunit, ATPase 4 (PSMC4), proteasome 26S subunit, non-ATPase 11 (PSMD11), and nuclear receptor corepressor 1 (NCOR1) (**Figure 1d**). The HC group had a total of 1316 DAPs, including 1234 upregulated and 82 downregulated proteins, compared with the NS and SA groups (**Figure 1e**).

### GO enrichment and KEGG pathway analysis

**Figure 2:**
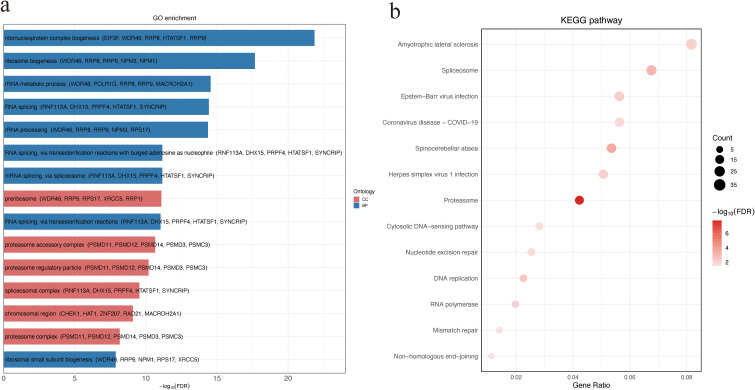
GO and KEGG enrichment analysis of DAPs between non-survivors and survivors. **(a)** GO enrichment of DAPs categorized by ontology. Bar plot showing the top 15 significantly enriched GO terms among DAPs. For each term, the top five associated DAPs are displayed in parentheses. **(b)** KEGG pathway enrichment analysis of DAPs. Bubble size indicates the number of genes enriched in each pathway, and color intensity reflects statistical significance (−log_10_ FDR). DAPs, differentially abundant proteins.

To explore the potential biological functions and pathways associated with the observed protein abundance differences, GO and KEGG enrichment analyses were performed. DAPs between NS and SA were significantly enriched for BP terms related to ribonucleoprotein complex and ribosome biogenesis, and for CC terms including the preribosome and proteasome accessory complex (**Figure 2a**; **Supplementary Table S1**). KEGG analysis highlighted several pathways, with amyotrophic lateral sclerosis (ALS) ranking highest by gene ratio(**Figure 2b**; **Supplementary Table S2**). These findings implicate perturbations in protein homeostasis and RNA/protein biogenesis in poor outcomes.

### Network-based clustering and KEGG pathway enrichment of candidate proteins

**Figure 3:**
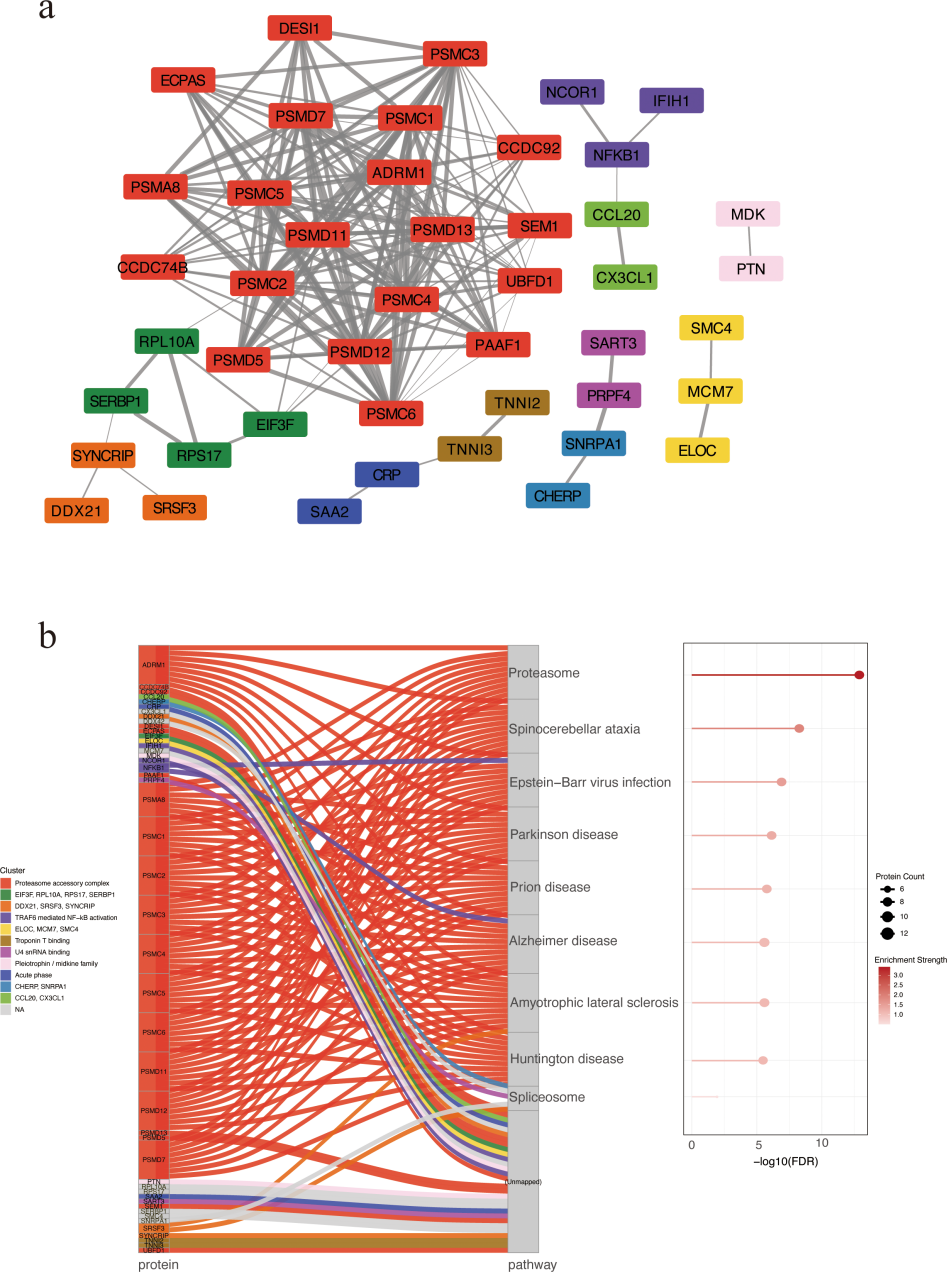
PPI network of candidate biomarkers. **(a)** PPI network revealing 11 protein clusters via MCL algorithm. Nodes represent proteins, and edges indicate predicted functional associations. Edge thickness corresponds to the STRING combined confidence score, with thicker edges reflecting higher confidence in PPIs. Each cluster is depicted in a distinct color. Unconnected nodes were excluded from the final visualization. **(b)** KEGG enrichment analysis by Sankey and bubble plots. Each flow represents the association between a specific protein cluster (left) and its significantly enriched KEGG pathway (right). Bubble size indicating the number of enriched proteins and color intensity reflecting enrichment significance. PPI, protein–protein interaction. MCL, Markov Clustering.

We constructed a STRING PPI network by integrating the top 50 proteins ranked by SHAP values from the XGBoost model and the top 50 proteins ranked by feature importance from the Random Forest classifier, yielding 85 nodes and 178 edges (clustering coefficient = 0.379; PPI enrichment *p* < 1.0 × 10^-^¹^6^). The resulting PPI network was clustered using the Markov Clustering (MCL) algorithm implemented in Cytoscape, revealing 11 distinct protein clusters (**Supplementary Table S3**). The most prominent cluster contained PSMD11 and multiple 26S proteasome subunits (PSMC4, PSMC2, PSMC3, PSMC6, PSMD7), pointing to coordinated proteasome involvement (**Figure 3a**). A Sankey plot mapped clusters to significantly enriched KEGG pathways, with several PSMD-containing clusters converging on the proteasome pathway (**Figure 3b**). These results suggest proteasome-associated pathways may play a central role in SFTS pathogenesis.

### Screening proteomic biomarkers via machine learning

**Figure 4:**
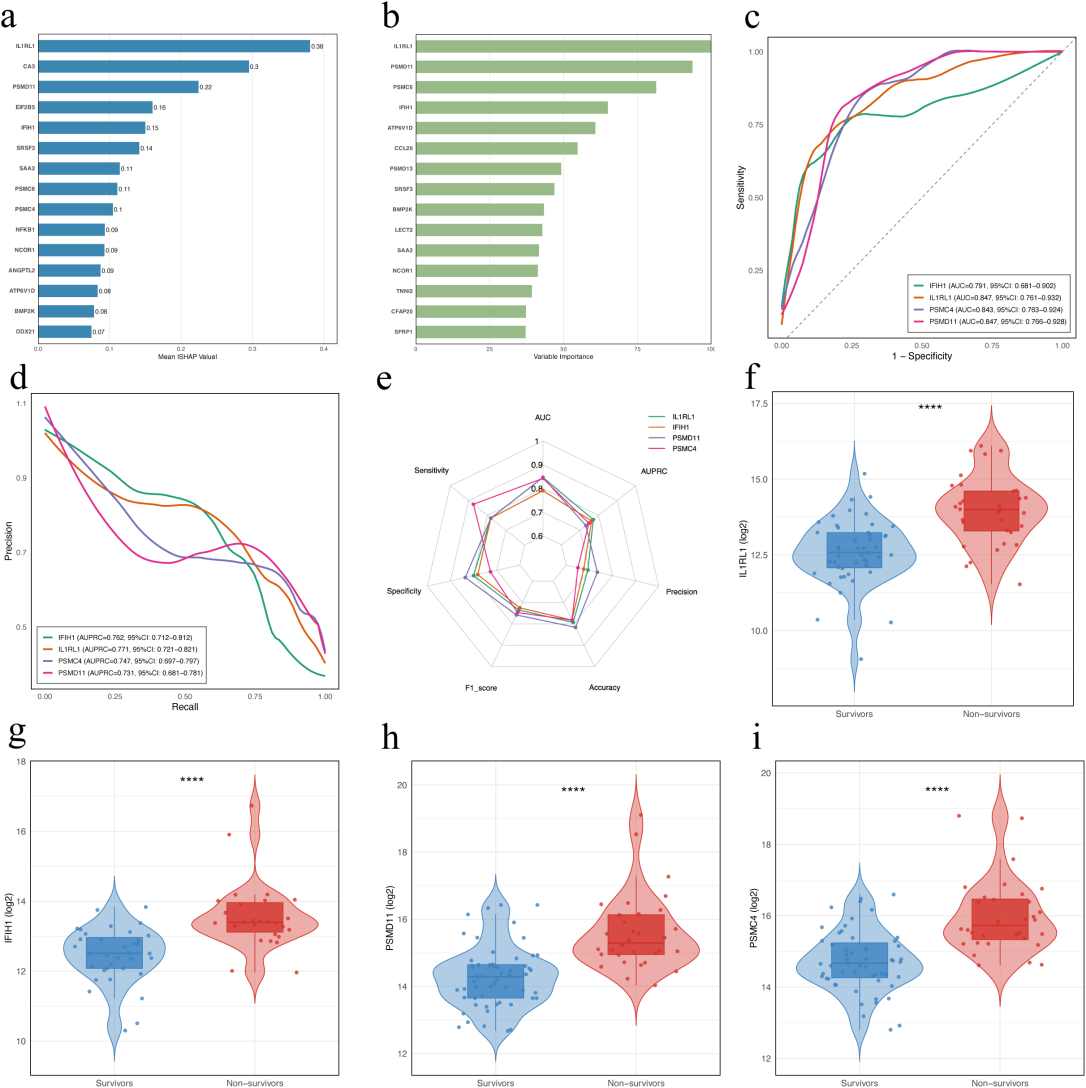
Identification and evaluation of potential prognostic biomarkers in patients with SFTS using machine learning. **(a)** Feature importance of DAPs in the XGBoost model. Bars indicate the mean absolute SHAP values averaged over 10-fold cross-validation. **(b)** Variable importance of proteins ranked by the Random Forest model. Variable importance scores were derived from a Random Forest classifier trained with 10-fold cross-validation, using DAPs (|log_2_FC| > 1, FDR < 0.05) as input features. **(c)** ROC curves for the four proteins. **(d)** Precision-recall (PR) curves for the four proteins. **(e)** Radar chart comparing diagnostic performance metrics of the four proteins. **(f–i)** Violin and box plots showing abundance levels of IL1RL1, IFIH1, PSMC4, and PSMD11 in survivors *vs*. non-survivors. Statistical significance was assessed using the Wilcoxon rank-sum test; **** indicates *p* < 0.0001. DAPs, differentially abundant proteins.

To explore potential prognostic biomarkers associated with poor outcomes in patients with SFTS, we trained XGBoost and Random Forest models using DAPs between the NS and SA. The XGBoost model interpreted via SHAP ranked IL1RL1, CA3, PSMD11, EIF2B5, IFIH1, SRSF3, SAA2, PSMC6, PSMC4, and NFKB1 as the top features (**Figure 4a**), while the Random Forest model highlighted IL1RL1, PSMD11, PSMC6, IFIH1, ATP6V1D, CCL20, PSMD13, SRSF3, BMP2K and LECT2 (**Figure 4b**). ROC analysis of four candidates (IL1RL1, PSMD11, PSMC4, IFIH1) showed AUCs of 0.847 (95% CI: 0.761–0.932), 0.847 (95% CI: 0.766–0.928), 0.843 (95% CI: 0.763–0.924), and 0.791 (95% CI: 0.681–0.902), respectively (**Figure 4c**). PR curves yielded AUPRCs of 0.771 (95% CI: 0.721–0.821), 0.731 (95% CI: 0.681–0.781), 0.747 (95% CI: 0.697–0.797), and 0.762 (95% CI: 0.712–0.812), respectively (**Figure 4d**). Comprehensive metric comparisons including AUC, AUPRC, sensitivity, specificity, precision, accuracy, and F1 score are shown in a radar chart (**Figure 4e**). Furthermore, violin plots demonstrated significantly elevated abundant of all four proteins in NS compared with SA (**Figures 4f–i**, *p* < 0.0001). Age-adjusted logistic regression analysis was performed to verify the robustness of these associations (**Supplementary Figure S1**).

### Correlation of candidate proteins with clinical indicators

**Figure 5:**
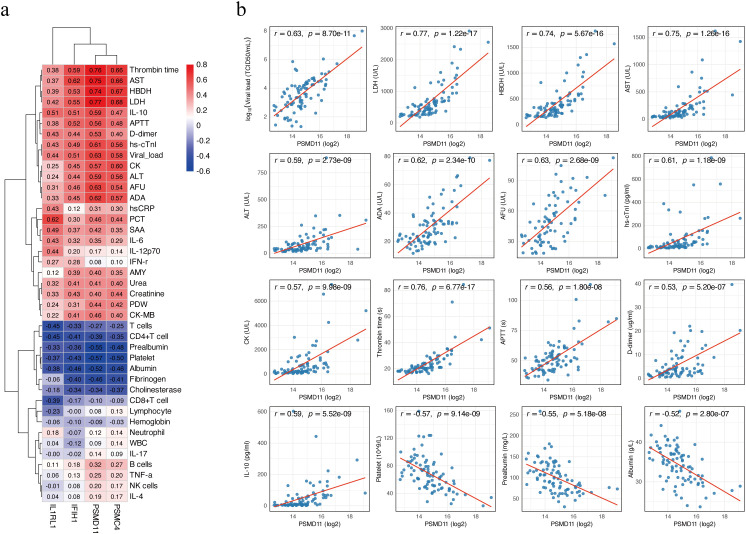
Correlation between candidate biomarkers and clinical parameters. **(a)** Heatmap of age-adjusted partial Spearman correlation coefficients between four proteins (PSMD11, IL1RL1, IFIH1, and PSMC4) and clinical laboratory parameters. Positive correlations are shown in red, and negative correlations in blue. **(b)** Scatter plots showing age-adjusted correlations between PSMD11 and clinical parameters. Viral load values were log_10_-transformed prior to analysis, while other variables were analyzed on their original scales. Partial Spearman correlation coefficients (*r*) and *p*-values are shown on each panel, and the red line represents the linear regression fit.

Age-adjusted partial Spearman correlation analysis was performed to assess associations between candidate protein levels and clinical laboratory parameters. Correlation heatmaps revealed broad associations of the four candidates (PSMD11, IL1RL1, IFIH1, PSMC4) with clinical laboratory parameters (**Figure 5a**). PSMD11 showed the strongest and most consistent relationships with hepatic dysfunction, myocardial injury, coagulation dysfunction, inflammation, and viral load. The strongest positive correlations were observed with LDH (*r* = 0.77), thrombin time (TT; *r* = 0.76), AST (*r* = 0.75), and hydroxybutyrate dehydrogenase (HBDH; *r* = 0.74). PSMD11 showed moderate to strong positive correlations with viral load (*r* = 0.63), alpha-L-fucosidase (AFU; *r* = 0.63), adenosine deaminase (ADA; *r* = 0.62), high-sensitivity cardiac troponin I (hs-cTnI; *r* = 0.61), IL-10 (*r* = 0.59), ALT (*r* = 0.59), creatine kinase (CK; *r* = 0.57), activated partial thromboplastin time (APTT; *r* = 0.56), and D-dimer (*r* = 0.53). Conversely, PSMD11 showed significant negative correlations with platelet count (*r* = −0.57), prealbumin (*r* = −0.55), and albumin (*r* = −0.52) (all *p* < 0.001; **Figure 5b**). These findings support a close linkage between PSMD11 and disease severity and multisystem pathophysiology in SFTS. Moreover, we performed a multivariable logistic regression model including age and these laboratory indicators, PSMD11 remained significantly associated with the outcome (OR = 4.20, 95% CI = 1.19–19.5, *p* = 0.039; **Supplementary Table S4**).

## Discussion

In this prospective clinical cohort, we employed DIA-based serum proteomics and integrated network-based analyses. By combining PPI mapping with MCL clustering, we delineated a coherent proteasome-associated module enriched in DAPs. Multiple lines of evidence from differential abundance, enrichment analyses, model explainability, and network mapping implicated a proteasome−centered axis as the dominant correlate of adverse outcomes in SFTS. This finding provides important mechanistic insight into how proteasome dysregulation may contribute to disease progression. To mitigate potential bias from the modest sample size and to reduce model overfitting, we further applied two complementary supervised ML algorithms-XGBoost and Random Forest for feature prioritization within DAPs and used interpretable feature-attribution metrics to rank their importance (25). Because age differed significantly between survivors and non-survivors, we further performed sensitivity analyses to account for this potential confounder. Four proteins (PSMD11, IL1RL1, IFIH1, and PSMC4) consistently exhibited the most pronounced and outcome-relevant alterations, with PSMD11 emerging as the most robust and stable signal. These results link proteomic alterations to clinical outcomes in SFTS and highlight PSMD11 and the proteasome pathway as promising candidates for prognostic assessment and potential translational research. In addition, this study extends current strategies for prognostic biomarker discovery in SFTS and highlights a set of novel candidate biomarkers that warrant further investigation. These key DAPs can serve not only as effective predictors of adverse outcomes but also as indicators of severe-related pathological mechanisms.

Previous studies have identified higher viral load, prolonged TT and APTT, as well as elevated levels of D-dimer, AST, ALT, LDH, CK, and cTnI as risk factors for adverse outcomes in patients with SFTS (26–28). Lower platelet count and albumin have also been linked to increased mortality (29). SFTS is characterized by multi-organ involvement and damage (30). While individual clinical laboratory indicators can typically reflect injury to only one or two specific organs, they fail to capture the systemic complexity of the disease. In our study, age-adjusted partial Spearman correlation analysis revealed that PSMD11 was significantly associated with a wide range of key clinical parameters, including markers of hepatic and myocardial injury, coagulation dysfunction, systemic inflammation, and viral load. These associations indicate that PSMD11 aligns with established clinical indicators of poor prognosis and may serve as a composite marker capturing multiple dimensions of disease severity in patients with SFTS.

PSMD11 is a non-ATPase lid subunit of the 19S regulatory particle that stabilizes 26S proteasome assembly and modulates substrate engagement (31). Ectopic abundance of PSMD11 increases proteasome assembly and activity in cells with relatively low basal proteasome function, whereas PSMD11 knockdown reduces the number of assembled proteasomes (32). PSMD11 serves as a dynamically regulated node that enhances 26S proteasome activity and maintains proteostasis under cellular stress (33). A recent study demonstrated that PSMD11 loss-of-function disrupts 26S proteasome assembly and triggers a persistent type I interferon signature through integrated stress response protein kinase R (34). In glomerular mesangial cells, miR-451 significantly inhibited inflammation and proliferation by downregulating PSMD11 and NF-κB p65, and transfection of miR-451 mimics significantly decreased levels of IL-1β, IL-6, and IL-8 (35). Consistently, our study also revealed significantly higher NF-κB abundance in non-survivors of SFTS. Previous studies have shown that an lncRNA, highly expressed in resting CD4+ T lymphocytes, recruits an HIV-1 regulatory protein to a PSMD11-containing ubiquitin–proteasome complex, thereby promoting its degradation and potentially contributing to HIV-1 latency (36). In addition, PSMD11 has been implicated in tumorigenesis partly through the modulation of tumor metabolism–related pathways. In hepatocellular carcinoma, its abundance is elevated and correlates with pathological stage and histologic grade (37). In lung carcinoma A549 cells, PSMD11 overexpression promotes proliferation, migration, invasion, and xenograft growth, while also altering immune-cell infiltration within the tumor microenvironment (38). In pancreatic ductal adenocarcinoma, PSMD11 and its related subunit PSMD14 are overexpressed, with higher abundance levels correlating with increased tumor malignancy and disease progression (39), and another study demonstrated that PSMD11 may serve as potential prognostic and diagnostic biomarkers in patients with early-stage disease (40). A previous study also reported that PSMD11 may play an important role in the metastasis of melanoma (41). Bortezomib is a potent proteasome inhibitor that has been widely used in the treatment of hematologic malignancies, acting through inhibition of the chymotrypsin-like site of the 20S proteolytic core within the 26S proteasome, which in turn induces cell-cycle arrest and apoptosis (42). Rather than being confined to a specific disease context, the dysregulation of PSMD11 appears to reflect broader cellular and immune processes. PSMD11 may be a multifunctional protein that contributes to the multi-organ dysfunction characteristic of severe SFTS, thereby underscoring its potential relevance to disease pathophysiology and prognosis.

This study has certain limitations. First, the sample size was relatively modest and derived from a single center, which may restrict the generalizability of our findings. Second, the lack of an independent external validation cohort limits the confirmation of robustness and the reproducibility of PSMD11 as a prognostic biomarker. Third, although PSMD11 emerged as a promising candidate, our results are associative and require functional validation to establish causality. Finally, only proteomic data were analyzed without integration of other omics layers or longitudinal sampling. Future multicenter studies with larger, externally validated cohorts and mechanistic experiments are warranted to confirm these findings and further elucidate the biological role of PSMD11 in SFTS.

In conclusion, our proteomic study identified a proteasome-centered axis associated with adverse outcomes in SFTS, with PSMD11 emerging as a prognostic biomarker closely linked to key clinical parameters and multi-organ dysfunction. These findings not only extend current strategies for biomarker discovery but also provide mechanistic insights into disease progression, thereby providing a foundation for future translational research and therapeutic exploration in SFTS.

## Data Availability

The datasets used and/or analyzed during the current study are available from the corresponding author on reasonable request.

## Glossary

SFTS: severe fever with thrombocytopenia syndrome
GO: Gene Ontology
KEGG: Kyoto Encyclopedia of Genes and Genomes
PPI: protein–protein interaction
ROC: receiver operating characteristic
DAPs: differentially abundant proteins
SFTSV: severe fever with thrombocytopenia syndrome virus
PHEIC: Public Health Emergency of International Concern
IL: interleukin
IFN: interferon
LDH: lactate dehydrogenase
CCL20: C-C motif chemokine 20
ML: machine learning
HC: healthy control
DIA: data-independent acquisition
RT-qPCR: reverse transcription quantitative polymerase chain reaction
LC-MS: liquid chromatography–mass spectrometry
KNN: k-nearest neighbors
IQR: interquartile range
FDR: false discovery rate FC, fold change
BP: biological process
CC: cellular component
MF: molecular function
STRING: search tool for retrieval of interacting genes/proteins
SD: standard deviation
PCA: principal component analysis
XGBoost: eXtreme Gradient Boosting
SHAP: SHapley Additive exPlanations
PR: precision-recall
AUC: area under the receiver operating characteristic curve
AUPRC: area under the precision-recall curve
CI: confidence intervals
WBC: white blood cell
ALT: alanine aminotransferase
AST: aspartate aminotransferase
MPV: mean platelet volume
TBil: total bilirubin
CIZ1: CDKN1A interacting zinc finger protein 1
PSMC4: proteasome 26S subunit, ATPase 4
PSMD11: proteasome 26S subunit, non-ATPase 11
NCOR1: nuclear receptor corepressor 1
ALS: amyotrophic lateral sclerosis
MCL: Markov Clustering
TT: thrombin time
HBDH: hydroxybutyrate dehydrogenase
ADA: adenosine deaminase
AFU: alpha-L-fucosidase
hs-cTnI: high-sensitivity cardiac troponin I
CK: creatine kinase
APTT: activated partial thromboplastin time.
