# Supplementary material for: Serum proteomics and machine learning identify PSMD11 as a prognostic biomarker in severe fever with thrombocytopenia syndrome
Source: Front Immunol. 2025 Nov 5;16:1693946. doi: 10.3389/fimmu.2025.1693946 (PMC12626957; doi:10.3389/fimmu.2025.1693946)
Supplement: Supplementary file 1 [file Table1.docx]

Supplementary

Supplementary Table S1. Top enriched GO terms of DAPs in cellular component between non-survivors and survivors.

| GO Term ID | GO_Term Description | Gene Symbol | Entrez Gene ID | log_2_(fold change) | Adjusted P value | Upregulated / Downregulated |
| --- | --- | --- | --- | --- | --- | --- |
| GO:0030684 | preribosome | WDR46 | 9277 | 3.0536 | 0.0174 | Upregulated |
| GO:0030684 | preribosome | RRP9 | 9136 | 3.7965 | 0.0381 | Upregulated |
| GO:0030684 | preribosome | RPS17 | 6218 | 3.9947 | 0.0144 | Upregulated |
| GO:0030684 | preribosome | XRCC5 | 7520 | 3.5544 | 0.0208 | Upregulated |
| GO:0030684 | preribosome | RRP1 | 8568 | 2.9523 | 0.0359 | Upregulated |
| GO:0030684 | preribosome | RPS14 | 6208 | 1.1198 | 0.0024 | Upregulated |
| GO:0030684 | preribosome | RPS23 | 6228 | 1.0792 | 0.0067 | Upregulated |
| GO:0030684 | preribosome | NOP14 | 8602 | 3.4113 | 0.0419 | Upregulated |
| GO:0030684 | preribosome | BYSL | 705 | 3.7412 | 0.0255 | Upregulated |
| GO:0030684 | preribosome | PWP2 | 5822 | 1.8529 | 0.0255 | Upregulated |
| GO:0030684 | preribosome | DNTTIP2 | 30836 | 3.7202 | 0.0023 | Upregulated |
| GO:0030684 | preribosome | NOP9 | 161424 | 4.7173 | 0.0309 | Upregulated |
| GO:0030684 | preribosome | FTSJ3 | 117246 | 4.3229 | 0.0020 | Upregulated |
| GO:0030684 | preribosome | EMG1 | 10436 | 3.5877 | 0.0072 | Upregulated |
| GO:0030684 | preribosome | LTV1 | 84946 | 1.9109 | 0.0256 | Upregulated |
| GO:0030684 | preribosome | MAK16 | 84549 | 2.2272 | 0.0406 | Upregulated |
| GO:0030684 | preribosome | WDR12 | 55759 | 4.7015 | 0.0028 | Upregulated |
| GO:0030684 | preribosome | NOL6 | 65083 | 3.7079 | 0.0488 | Upregulated |
| GO:0030684 | preribosome | PPAN | 56342 | 1.4927 | 0.0472 | Upregulated |
| GO:0030684 | preribosome | NOB1 | 28987 | 2.7267 | 0.0241 | Upregulated |
| GO:0030684 | preribosome | DIMT1 | 27292 | 2.3427 | 0.0084 | Upregulated |
| GO:0030684 | preribosome | RRP7A | 27341 | 1.7041 | 0.0256 | Upregulated |
| GO:0030684 | preribosome | NOC2L | 26155 | 3.5892 | 0.0194 | Upregulated |
| GO:0030684 | preribosome | UTP18 | 51096 | 2.0795 | 0.0407 | Upregulated |
| GO:0022624 | proteasome accessory complex | PSMD11 | 5717 | 1.3147 | 0.0002 | Upregulated |
| GO:0022624 | proteasome accessory complex | PSMD12 | 5718 | 1.3749 | 0.0173 | Upregulated |
| GO:0022624 | proteasome accessory complex | PSMD14 | 10213 | 1.4016 | 0.0235 | Upregulated |
| GO:0022624 | proteasome accessory complex | PSMD3 | 5709 | 1.1966 | 0.0004 | Upregulated |
| GO:0022624 | proteasome accessory complex | PSMC3 | 5702 | 1.0838 | 0.0005 | Upregulated |
| GO:0022624 | proteasome accessory complex | PSMC2 | 5701 | 1.1931 | 0.0002 | Upregulated |
| GO:0022624 | proteasome accessory complex | PSMC4 | 5704 | 1.2515 | 0.0001 | Upregulated |
| GO:0022624 | proteasome accessory complex | PSMD7 | 5713 | 1.0930 | 0.0004 | Upregulated |
| GO:0022624 | proteasome accessory complex | PSME3 | 10197 | 4.1514 | 0.0351 | Upregulated |
| GO:0022624 | proteasome accessory complex | PSMC1 | 5700 | 1.1104 | 0.0004 | Upregulated |
| GO:0022624 | proteasome accessory complex | PSMC5 | 5705 | 1.0785 | 0.0004 | Upregulated |
| GO:0022624 | proteasome accessory complex | PSMC6 | 5706 | 1.2068 | 0.0004 | Upregulated |
| GO:0022624 | proteasome accessory complex | PSMD13 | 5719 | 1.1870 | 0.0005 | Upregulated |

Supplementary Table S2. Top enriched KEGG pathways of DAPs between non-survivors and survivors.

| KEGG Pathway ID | KEGG Pathway Description | Gene Symbol | Entrez Gene ID | log_2_(fold change) | Adjusted P value | Upregulated / Downregulated |
| --- | --- | --- | --- | --- | --- | --- |
| hsa05014 | Amyotrophic lateral sclerosis | PSMD11 | 5717 | 1.3147 | 0.0002 | Upregulated |
| hsa05014 | Amyotrophic lateral sclerosis | PSMD12 | 5718 | 1.3749 | 0.0173 | Upregulated |
| hsa05014 | Amyotrophic lateral sclerosis | PSMD14 | 10213 | 1.4016 | 0.0235 | Upregulated |
| hsa05014 | Amyotrophic lateral sclerosis | PSMD3 | 5709 | 1.1966 | 0.0004 | Upregulated |
| hsa05014 | Amyotrophic lateral sclerosis | PSMC3 | 5702 | 1.0838 | 0.0005 | Upregulated |
| hsa05014 | Amyotrophic lateral sclerosis | PSMB1 | 5689 | 2.4123 | 0.0452 | Upregulated |
| hsa05014 | Amyotrophic lateral sclerosis | PSMB4 | 5692 | 3.0139 | 0.0337 | Upregulated |
| hsa05014 | Amyotrophic lateral sclerosis | PSMC2 | 5701 | 1.1931 | 0.0002 | Upregulated |
| hsa05014 | Amyotrophic lateral sclerosis | MATR3 | 9782 | 1.2044 | 0.0337 | Upregulated |
| hsa05014 | Amyotrophic lateral sclerosis | PSMC4 | 5704 | 1.2515 | 0.0001 | Upregulated |
| hsa05014 | Amyotrophic lateral sclerosis | NUP153 | 9972 | 3.7566 | 0.0165 | Upregulated |
| hsa05014 | Amyotrophic lateral sclerosis | PSMD7 | 5713 | 1.0930 | 0.0004 | Upregulated |
| hsa05014 | Amyotrophic lateral sclerosis | PSMC1 | 5700 | 1.1104 | 0.0004 | Upregulated |
| hsa05014 | Amyotrophic lateral sclerosis | PSMC5 | 5705 | 1.0785 | 0.0004 | Upregulated |
| hsa05014 | Amyotrophic lateral sclerosis | PSMC6 | 5706 | 1.2068 | 0.0004 | Upregulated |
| hsa05014 | Amyotrophic lateral sclerosis | SRSF3 | 6428 | 1.1147 | 0.0011 | Upregulated |
| hsa05014 | Amyotrophic lateral sclerosis | SQSTM1 | 8878 | 3.5076 | 0.0482 | Upregulated |
| hsa05014 | Amyotrophic lateral sclerosis | DCTN2 | 10540 | 3.5191 | 0.0452 | Upregulated |
| hsa05014 | Amyotrophic lateral sclerosis | SRSF7 | 6432 | 1.5874 | 0.0421 | Upregulated |
| hsa05014 | Amyotrophic lateral sclerosis | NUP54 | 53371 | 3.8044 | 0.0459 | Upregulated |
| hsa05014 | Amyotrophic lateral sclerosis | NUP133 | 55746 | 1.5039 | 0.0486 | Upregulated |
| hsa05014 | Amyotrophic lateral sclerosis | ATXN2L | 11273 | 5.4566 | 0.0104 | Upregulated |
| hsa05014 | Amyotrophic lateral sclerosis | OPTN | 10133 | 2.3873 | 0.0383 | Upregulated |
| hsa05014 | Amyotrophic lateral sclerosis | PIK3R4 | 30849 | 3.3443 | 0.0110 | Upregulated |
| hsa05014 | Amyotrophic lateral sclerosis | DCTN5 | 84516 | 4.7463 | 0.0072 | Upregulated |
| hsa05014 | Amyotrophic lateral sclerosis | NUP58 | 9818 | 3.6050 | 0.0354 | Upregulated |
| hsa05014 | Amyotrophic lateral sclerosis | TUBB1 | 81027 | -1.3852 | 0.0072 | Downregulated |
| hsa05014 | Amyotrophic lateral sclerosis | UBQLN1 | 29979 | 4.1082 | 0.0348 | Upregulated |
| hsa05014 | Amyotrophic lateral sclerosis | PSMD13 | 5719 | 1.1870 | 0.0005 | Upregulated |
| hsa03040 | Spliceosome | DHX15 | 1665 | 1.2643 | 0.0016 | Upregulated |
| hsa03040 | Spliceosome | PRPF4 | 9128 | 4.3828 | 0.0133 | Upregulated |
| hsa03040 | Spliceosome | SNRPA | 6626 | 3.5426 | 0.0494 | Upregulated |
| hsa03040 | Spliceosome | SNRPA1 | 6627 | 3.5444 | 0.0115 | Upregulated |
| hsa03040 | Spliceosome | EIF4A3 | 9775 | 1.7029 | 0.0417 | Upregulated |
| hsa03040 | Spliceosome | RBM25 | 58517 | 3.0863 | 0.0472 | Upregulated |
| hsa03040 | Spliceosome | SNRPE | 6635 | 1.2920 | 0.0421 | Upregulated |
| hsa03040 | Spliceosome | SNRPF | 6636 | 3.7542 | 0.0108 | Upregulated |
| hsa03040 | Spliceosome | SNRPD1 | 6632 | 1.7767 | 0.0402 | Upregulated |
| hsa03040 | Spliceosome | SRSF3 | 6428 | 1.1147 | 0.0011 | Upregulated |
| hsa03040 | Spliceosome | SRSF2 | 6427 | 2.2903 | 0.0202 | Upregulated |
| hsa03040 | Spliceosome | SRSF9 | 8683 | 3.7333 | 0.0244 | Upregulated |
| hsa03040 | Spliceosome | SRSF5 | 6430 | 4.0915 | 0.0085 | Upregulated |
| hsa03040 | Spliceosome | SRSF6 | 6431 | 1.3643 | 0.0012 | Upregulated |
| hsa03040 | Spliceosome | DHX8 | 1659 | 5.0096 | 0.0074 | Upregulated |
| hsa03040 | Spliceosome | SF3B4 | 10262 | 3.5953 | 0.0241 | Upregulated |
| hsa03040 | Spliceosome | SRSF7 | 6432 | 1.5874 | 0.0421 | Upregulated |
| hsa03040 | Spliceosome | DDX42 | 11325 | 1.3975 | 0.0172 | Upregulated |
| hsa03040 | Spliceosome | CHERP | 10523 | 5.4344 | 0.0039 | Upregulated |
| hsa03040 | Spliceosome | PRPF38A | 84950 | 2.9371 | 0.0337 | Upregulated |
| hsa03040 | Spliceosome | PRPF31 | 26121 | 3.5940 | 0.0421 | Upregulated |
| hsa03040 | Spliceosome | ACIN1 | 22985 | 1.1564 | 0.0402 | Upregulated |
| hsa03040 | Spliceosome | WBP11 | 51729 | 4.0114 | 0.0174 | Upregulated |

Supplementary Table S3. 11 distinct protein clusters by Markov Clustering

| Cluster Number | Description | Gene Count | Protein Name | Protein Identifier |
| --- | --- | --- | --- | --- |
| 1 | Proteasome accessory complex | 20 | ADRM1 | 9606.ENSP00000478877 |
| 1 | Proteasome accessory complex | 20 | CCDC74B | 9606.ENSP00000308873 |
| 1 | Proteasome accessory complex | 20 | CCDC92 | 9606.ENSP00000238156 |
| 1 | Proteasome accessory complex | 20 | DESI1 | 9606.ENSP00000263256 |
| 1 | Proteasome accessory complex | 20 | ECPAS | 9606.ENSP00000259335 |
| 1 | Proteasome accessory complex | 20 | PAAF1 | 9606.ENSP00000438071 |
| 1 | Proteasome accessory complex | 20 | PSMA8 | 9606.ENSP00000311121 |
| 1 | Proteasome accessory complex | 20 | PSMC1 | 9606.ENSP00000261303 |
| 1 | Proteasome accessory complex | 20 | PSMC2 | 9606.ENSP00000391211 |
| 1 | Proteasome accessory complex | 20 | PSMC3 | 9606.ENSP00000481029 |
| 1 | Proteasome accessory complex | 20 | PSMC4 | 9606.ENSP00000157812 |
| 1 | Proteasome accessory complex | 20 | PSMC5 | 9606.ENSP00000310572 |
| 1 | Proteasome accessory complex | 20 | PSMC6 | 9606.ENSP00000484998 |
| 1 | Proteasome accessory complex | 20 | PSMD11 | 9606.ENSP00000261712 |
| 1 | Proteasome accessory complex | 20 | PSMD12 | 9606.ENSP00000348442 |
| 1 | Proteasome accessory complex | 20 | PSMD13 | 9606.ENSP00000396937 |
| 1 | Proteasome accessory complex | 20 | PSMD5 | 9606.ENSP00000210313 |
| 1 | Proteasome accessory complex | 20 | PSMD7 | 9606.ENSP00000219313 |
| 1 | Proteasome accessory complex | 20 | SEM1 | 9606.ENSP00000481021 |
| 1 | Proteasome accessory complex | 20 | UBFD1 | 9606.ENSP00000379217 |
| 2 | EIF3F, RPL10A, RPS17, SERBP1 | 4 | EIF3F | 9606.ENSP00000431800 |
| 2 | EIF3F, RPL10A, RPS17, SERBP2 | 4 | RPL10A | 9606.ENSP00000363018 |
| 2 | EIF3F, RPL10A, RPS17, SERBP3 | 4 | RPS17 | 9606.ENSP00000498019 |
| 2 | EIF3F, RPL10A, RPS17, SERBP4 | 4 | SERBP1 | 9606.ENSP00000360034 |
| 3 | TRAF6 mediated NF-kB activation | 3 | IFIH1 | 9606.ENSP00000497271 |
| 3 | TRAF6 mediated NF-kB activation | 3 | NCOR1 | 9606.ENSP00000268712 |
| 3 | TRAF6 mediated NF-kB activation | 3 | NFKB1 | 9606.ENSP00000226574 |
| 3 | DDX21, SRSF3, SYNCRIP | 3 | DDX21 | 9606.ENSP00000346120 |
| 3 | DDX21, SRSF3, SYNCRIP | 3 | SRSF3 | 9606.ENSP00000362820 |
| 3 | DDX21, SRSF3, SYNCRIP | 3 | SYNCRIP | 9606.ENSP00000358635 |
| 4 | ELOC, MCM7, SMC4 | 3 | ELOC | 9606.ENSP00000478121 |
| 4 | ELOC, MCM7, SMC5 | 3 | MCM7 | 9606.ENSP00000307288 |
| 4 | ELOC, MCM7, SMC6 | 3 | SMC4 | 9606.ENSP00000341382 |
| 6 | Troponin T binding | 2 | TNNI2 | 9606.ENSP00000371336 |
| 6 | Troponin T binding | 2 | TNNI3 | 9606.ENSP00000341838 |
| 7 | U4 snRNA binding | 2 | PRPF4 | 9606.ENSP00000363313 |
| 7 | U4 snRNA binding | 2 | SART3 | 9606.ENSP00000449386 |
| 8 | Pleiotrophin / midkine family | 2 | MDK | 9606.ENSP00000385451 |
| 8 | Pleiotrophin / midkine family | 2 | PTN | 9606.ENSP00000341170 |
| 9 | Acute phase | 2 | CRP | 9606.ENSP00000255030 |
| 9 | Acute phase | 2 | SAA2 | 9606.ENSP00000436126 |
| 10 | CHERP, SNRPA1 | 2 | CHERP | 9606.ENSP00000439856 |
| 10 | CHERP, SNRPA2 | 2 | SNRPA1 | 9606.ENSP00000254193 |
| 11 | CCL20, CX3CL1 | 2 | CCL20 | 9606.ENSP00000351671 |
| 11 | CCL20, CX3CL2 | 2 | CX3CL1 | 9606.ENSP00000006053 |

Supplementary Table S4. Multivariable logistic regression analysis of the clinical outcome including PSMD11 and clinical laboratory parameters

|  | OR | 95%CI | *p*-value |
| --- | --- | --- | --- |
| PSMD11 | 4.20 | 1.19-19.5 | 0.0389 |
| Age | 1.06 | 0.979-1.15 | 0.179 |
| Viral load (log_10_) | 2.66 | 1.12-7.58 | 0.040 |
| LDH | 0.998 | 0.985-1.01 | 0.778 |
| HBDH | 1.01 | 0.988-1.03 | 0.558 |
| AST | 1.00 | 0.991-1.01 | 0.742 |
| ADA | 0.88 | 0.764-0.982 | 0.040 |
| AFU | 0.992 | 0.949-1.04 | 0.731 |
| hs-cTnI | 1.01 | 0.996-1.02 | 0.231 |
| Thrombin time | 1.01 | 0.892-1.24 | 0.853 |

Hosmer–Lemeshow goodness-of-fit test: X² = 6.367, *p* = 0.606. The model included age and eight laboratory parameters in addition to PSMD11.

Abbreviations: OR, odds ratio; CI, confidence interval; LDH, lactate dehydrogenase; HBDH, α-hydroxybutyrate dehydrogenase; AST, aspartate aminotransferase; ADA, adenosine deaminase; AFU, α-fucosidase; hs-cTnI, high-sensitivity cardiac troponin I.


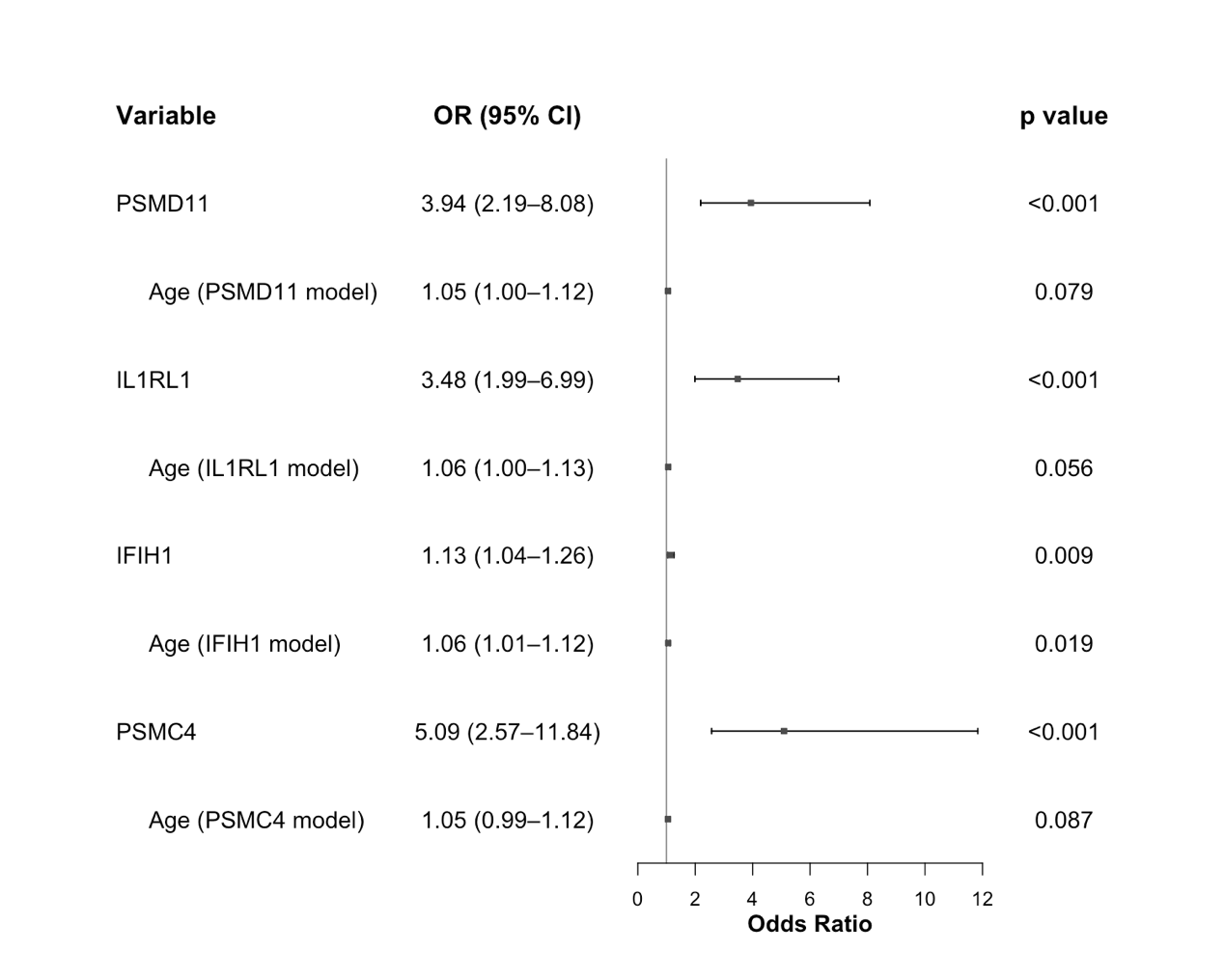


Supplementary Figure S1. Forest plot of multivariable logistic regression adjusted for age. Forest plot showing age-adjusted odds ratios (OR) and 95% confidence intervals (CI) from multivariable logistic regression analyses evaluating the association between four candidate proteins (PSMD11, IL1RL1, IFIH1, and PSMC4) and mortality in patients with SFTS. Each model included one protein and age as covariates.
